# Mid age *APOE* ε4 carriers show memory-related functional differences and disrupted structure-function relationships in hippocampal regions

**DOI:** 10.1038/s41598-020-59272-0

**Published:** 2020-02-20

**Authors:** Simon L. Evans, Nicholas G. Dowell, Fenella Prowse, Naji Tabet, Sarah L. King, Jennifer M. Rusted

**Affiliations:** 1 School of Psychology and Sussex Neuroscience, University of Sussex, Brighton, East Sussex BN1 9QG United Kingdom; 2 School of Psychology, Faculty of Health and Medical Sciences, University of Surrey, Guildford, Surrey GU2 7XH United Kingdom; 3 Clinical Imaging Sciences Centre, Brighton and Sussex Medical School, Brighton, East Sussex BN1 9RR United Kingdom; 4 Brighton and Sussex Medical School (BSMS), Brighton, East Sussex BN1 9RN United Kingdom; 5 Centre for Dementia Studies, Brighton and Sussex Medical School, Brighton, East Sussex BN1 9RN United Kingdom

**Keywords:** Alzheimer's disease, Neural ageing

## Abstract

Carriers of the APOE e4 allele are at higher risk of age-related cognitive decline and Alzheimer’s disease (AD). The underlying neural mechanisms are uncertain, but genotype differences in medial temporal lobe (MTL) functional activity and structure at mid-age might contribute. We tested 16 non-e4 and 16 e4 carriers (aged 45–55) on a subsequent memory task in conjunction with MRI to assess how hippocampal volume (from T1 structural) and microstructure (neurite orientation-dispersion, from NODDI) differs by genotype and in relation to memory encoding. No previous study has investigated APOE effects on hippocampal microstructure using NODDI. Recall performance did not differ by genotype. A genotype by condition interaction in left parahippocampus indicated that in e4 carriers activity did not differentiate subsequently remembered from forgotten words. Hippocampal volumes and microstructure also did not differ by genotype but hippocampal volumes correlated positively with recognition performance in non-e4 carriers only. Similarly, greater hippocampal neurite orientation-dispersion was linked to better recall but only in non-e4s. Thus, we suggest that mid-age e4 carriers show a breakdown of normal MTL activation and structure-performance relationships. This could reflect an inability to utilise compensatory mechanisms, and contribute to higher risk of cognitive decline and AD in later life.

## Introduction

In humans, the Apolipoprotein E (*APOE*) gene has 3 variants (e2, e3, e4); the e4 allele has been subject to considerable research interest due to it being a well-established risk factor for late-onset Alzheimer’s disease (AD)^1^ with e4 carriers being approximately 3 times more likely to develop AD relative to e3 carriers^2^. The e4 allele also affects cognitive ageing trajectories in healthy individuals. Healthy older e4 carriers (from this point referred to as e4+) perform worse than non-e4 carriers (e4−) on measures of episodic memory, executive functioning and overall global cognitive ability^3^. Longitudinal studies in healthy populations point to more rapid age-related cognitive decline in e4+^4,5^. Small detrimental effects of e4 on cognition appear to be evident at mid age^4,6^ with these becoming more pronounced beyond the 6^th^ decade^7^. However, midlife effects of *APOE* are not well studied, despite evidence that risk factor exposure during this life stage significantly influences AD risk in later life^8^. With accumulation of AD–related neuropathology beginning some decades before symptom onset^9^, midlife thus likely represents the optimal opportunity to intervene against cognitive decline and AD.

Brain structural differences have been detected in healthy e4 carriers. Imaging studies have tended to focus on the medial temporal lobe (MTL) since this structure is amongst the first to evidence AD-related pathology^10^. Reduced hippocampal volumes have been reported in healthy older e4+^11,12^ but studies in younger samples tend to report no volumetric differences e.g.^13–15^. MTL functional activation differences have been reported in younger adults however, often in the form of overactivation in e4+ relative to e4− peers. Using a subsequent memory paradigm (in which an acquisition phase containing a set of stimuli to be remembered is presented, followed by a subsequent recognition test where those same stimuli are presented again, interleaved with some novel stimuli), Dennis *et al*.^16^ reported significantly greater MTL activation to subsequently remembered items in e4+ aged 20–25, although recognition performance was matched. Likewise, we have shown increased activity in left MTL to subsequently remembered words in young adult e4+ with no differences in recognition performance^17^. Filippini *et al*.^13^ found that young adult e4+ (mean age 28) show a pattern of hippocampal over-recruitment in the recognition phase of a subsequent memory paradigm, in a task using non-verbal stimuli. MTL activity has also been reported in young adult e4+ during tasks not expected to engage that region, e.g. a Stroop task^18^ and a covert attention task^19^, and MTL coactivation differences have been shown in the resting state^13^. These findings have been interpreted as representing a compensatory mechanism whereby young adult e4+ recruit additional neural resources to maintain cognitive performance^20^.

In contrast, at mid age there is evidence that hippocampal activation is instead reduced in e4+. Trivedi *et al.*^21^ found that healthy mid age e4+ (mean age 54, who had at least one parent with AD) show reduced hippocampal activity during subsequent memory recognition. There were no differences in cognitive performance or MTL volumes, but performance on a verbal learning task correlated with MTL activation in e4− only. This suggests that the relationship between learning performance and MTL activity is disrupted in mid age e4+. Reduced MTL activity has also been reported in older (mean age 73) cognitively normal e4+ during encoding of visuospatial memory, in the absence of any performance differences. Again, MTL encoding activity correlated with performance in e4− only^22^. This is not attributable to a global decrease in e4+ neural activity, since follow-up work using the same task in a slightly younger age group (mean age 64) found that activity in MTL but not visual cortex was reduced in e4+^23^. These findings point to a specific downregulation of hippocampal activity in mid age and older e4+, and a breakdown of normal function-performance relationships.

Cross-sectional studies examining effects of age range add further evidence. Contrasting younger (20–35 yrs) and older (50–78) age groups, Filippini *et al*.^24^ found an age by genotype interaction in MTL activity during presentation of novel vs. familiar images: activity decreased with age in e4+ but increased in e4−, there were no performance or MTL volumetric differences. Likewise, in a sample aged 61–80, only e4+ showed a decrease in MTL activation with age during a working memory task, again no performance or brain volume differences were seen^25^. While MTL overactivity might characterise young adult e4+, these studies suggest that this effect is reversed perhaps as early as mid age. This could reflect early MTL vulnerability in this group, evident well before cognitive deficits become apparent. This would be consistent with evidence from functional and diffusion imaging studies suggesting accelerated neural ageing in e4+^25,26^, detectable at mid age^27^.

Conflicting results should be noted, however. Older e4+ have also been shown to overactivate MTL and other regions in response to picture encoding^28^, recall of learnt word pairs^29^ and object/context encoding^30^. Choice of task could be a factor. Evidence suggests that, in older adults, activation levels during semantic memory tasks predict cognitive decline better than episodic memory tasks^31^. However recent work has shown that in older adults, hippocampal overactivation during episodic memory recall is associated with higher levels of tau in cerebrospinal fluid^32^. Since higher levels of tau in cerebrospinal fluid have been shown in older e4+^33^, this association could contribute to MTL overactivity in older e4+. Amyloid pathology could also be important since cerebral amyloid levels have been shown to be higher in healthy mid-age and older e4+, particularly in those with increased hippocampal activity^34,35^. It is worth noting that none of the aforementioned studies considered e4 dose effects due to insufficient numbers of homozygous e4 carriers being recruited, precluding stratification. Homozygous carriers of the e4 allele (where present) were included alongside those heterozygous for e4 to form an “e4 carrier” group. Most (but not all) studies excluded carriers of the e2 allele. Work by Cacciaglia and colleagues has examined e4 dose effects in a healthy mid-age sample (mean age 58), finding that while e4 dose did not affect cognitive performance, it did affect relationships between episodic memory performance and grey matter volumes (including in MTL)^36^, in a manner suggesting accelerated ageing in e4+; e4 dose was also associated with reduced volume in various brain regions including right hippocampus^37^.

In this study, we sought to better characterize effects of APOE genotype status on MTL function and structure. We focused on ‘mid age’, recruiting participants within a narrow age range (45–55 years). This represents a critical life stage in terms of AD risk but is currently understudied. Mid age is likely the most informative age point in terms of detecting pathological changes that occur prior to detrimental effects on cognition, which can confound interpretations. This life stage also likely represents the optimal ‘window’ for monitoring and intervention in e4+^38^.

Participants performed a subsequent memory task (functional magnetic resonance imaging (fMRI) during the acquisition phase) to assess functional activation to successfully encoded vs. subsequently forgotten items. The task used incidental encoding: in the acquisition phase participants were presented with a series of words and asked to make a classification response. In the subsequent recognition phase, all words presented at acquisition were presented again, interleaved with novel words: participants responded ‘old’ or ‘new’ to each. MTL activity to an item during incidental encoding predicts subsequent recognition success^39^. Based on the literature we predicted reduced MTL activity in mid age e4+ and an a priori region of interest (ROI) approach was taken. The ROI mask encompassed hippocampus and parahippocampus. In e4+, we predicted reduced encoding-related activity within this ROI, indicating early MTL vulnerability. Based on previous findings, we did not expect e4+ to show memory performance deficits.

T1 structural images were also acquired to test for genotype differences in hippocampal volumes. Genotype effects on relationships between volumes and memory performance were also explored since previous studies point to disrupted relationships between MTL neural markers and memory performance in e4+^21,22^. Relationships between white matter integrity and episodic memory also appear to be disrupted in healthy older e4+^40^. On this basis, we predicted that the normal MTL structure-function relationships observed in healthy individuals^41^ would be disrupted in e4+, with e4+ showing a breakdown in the relationship between hippocampal volume and memory performance measures.

A major strength of this study was the inclusion of neurite orientation dispersion and density imaging (NODDI). NODDI is an advanced diffusion MRI technique which is capable of detecting microstructural properties of the dentrites and axons in the brain^42^. Previous work has used DTI to investigate APOE effects on microstructure in healthy individuals but this is the first study to employ NODDI to address this question. Previous DTI results have been mixed. In young adult e4+, one study reported increased fractional anisotropy (FA), particularly in parahippocampal cingulum bundle^43^. Others have reported FA to be decreased^44^ or no different^45^. At mid-age, increased radial diffusivity (but no differences in FA) were found in e4 homozygotes^46^. In heathy older e4+, decreased FA has been reported in the left parahippocampal white matter^47^ and other white matter tracts^48^ although other studies have not replicated these^40,49^. The NODDI model offers several advantages over DTI. Unlike DTI, which assumes that each voxel contains a single tissue compartment with Gaussian diffusion, NODDI uses a three-compartment model that represents three distinct microstructural environments for each voxel – intracellular, extracellular and CSF. It differentiates two of the key contributing variables to FA by providing estimates of both neurite density and neurite orientation dispersion. Compared to DTI, the NODDI model better accounts for the confounding effects of free-water contamination, and has been shown to be superior in detecting clinically-relevant microstructural changes^50^ although some shortcomings should be noted. In particular, the technique assumes a fixed value for intrinsic diffusivity across the brain. If this assumption is violated it could lead to a bias in the extracted parameters^51,52^.

NODDI has recently been used to show that e4+ with early onset AD have different patterns of white matter neurodegeneration^53^, but has not previously been used to explore APOE effects in healthy individuals. As we were focusing on effects in hippocampus/parahippocampus, we examined how neurite orientation dispersion (orientation dispersion index (ODI), the degree of dispersion of the fibre orientations) within MTL might differ by genotype. This was motivated by evidence that hippocampal ODI has a neuroprotective role, which we hypothesised might be lacking in e4+. In healthy individuals, higher ODI in bilateral hippocampus has been shown to protect against age-related differences in cognitive performance, this was the only region in which ODI exerted such an effect^54^. We hypothesised that differences in ODI could contribute to enhanced risk of poorer cognitive ageing and cognitive decline in e4+. ODI in bilateral MTL was therefore examined in terms of genotype effects and links to memory performance. We again predicted that relationships between memory performance and this structural measure would be disrupted in e4+. If so, this would add valuable new information regarding underlying mechanisms.

## Materials and Methods

### Participants

All participants volunteered under a written informed consent procedure. Ethical approval was granted by the Sussex University Schools of Psychology and Life Sciences Research Ethics Committee. All relevant guidelines and regulations were followed throughout. In the initial screening phase 165 healthy volunteers were recruited via advertisements at local clubs, universities and community centres, as part of a wider program of work investigating APOE behavioural effects in this age group^55,56^. The following inclusion criteria were employed: age 45–55, fluent English speaker, right handed, MRI-safe, no diagnosis of high blood pressure, and not currently being treated for a psychiatric condition. The genotyping procedure has been described previously^17^: *APOE* genotype was determined by buccal swab and Genotype analyses were performed by a third party (LGC Genomics, Hoddesdon, UK) using fluorescence-based competitive allele-specific polymerase chain reaction (KASPar) targeting two APOE single-nucleotide polymorphisms (SNPs): rs429358 and rs7412. Protocols specified by the Human Tissue Act were followed throughout, participants consented to not being informed of their genotyping result, and volunteer call-back was performed by a third party so that the researcher remained blind. Invitation to take part in this study was based on a random sampling so genotype status could not be inferred from an invitation to take part. fMRI power calculation: We performed a power analysis using fMRIpower software (fMRIpower.org) developed by Mumford *et al*.^57^. This toolbox estimates the power to detect significant activation within specific ROIs, based on previous results. An appropriate group analysis (genotype effects during a prospective memory task, bilateral superior parietal lobe as ROI) was entered into the toolbox, from data we have collected and published previously in mid-age e4+ and e4−, which used the same scanner and acquisition protocol, and a comparable design/analytical model^27^. The results from the power analysis were that testing 15 participants would provide at least 80% power to detect a significant difference between-groups with a corrected p-value of p < 0.05. Amongst those invited and subsequently consented to take part: 16 were e3/e3 carriers (these comprised the e4− group); 11 were e3/e4 and 5 were e4/e4 (these comprised the e4+ group, totaling 16); 1 participant was an e2/e4 and excluded from analyses based on evidence that the e2 allele might be protective^58^. Thus the final sample consisted of 2 groups: 16 e4− and 16 e4+. The Montreal Cognitive Assessment (MoCA^59^) was administered to ascertain the cognitive status of all participants. No participant scored below 26 on this measure.

Between the two groups, there were no significant differences in age, gender balance, NART IQ, MoCA score or years of education (Table 1). Nevertheless in the imaging analyses, gender was entered as a covariate.

**Table 1:** Volunteer characteristics by genotype group. P value (2-tailed) from chi squared test (Gender) or between-group t-tests (all other measures).

Group	Age (yrs, s.d.)	Gender	NART IQ (s.d.)	MoCA score (/30, s.d.)	Education (yrs, s.d.)
e4− (n = 16)	50.1 ± 2.8	11 F/5M	120.9 ± 3.3	28.9(1.5)	17.0 ± 2.0
e4+ (n = 16)	50.3 ± 3.8	9 F/7 M	118.1 ± 4.6	27.8(1.3)	17.8 ± 4.1
p value	0.877	0.465	0.071	0.078	0.489

### Subsequent memory task

**Figure 1:**
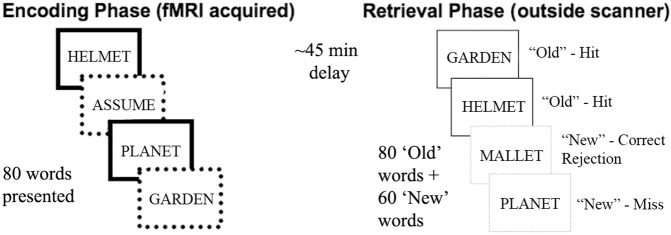
Task schematic.

The task has been described elsewhere^17^: it was constructed and run using MATLAB as a component of a one hour scanner session. The acquisition phase of the task was presented as a semantic categorisation task, and consisted of 80 words (all of which were 6 letters long) presented sequentially. Each word was presented at a central point on-screen for 1 second. There was a variable ISI of 2.5 to 4.5 seconds. A mask (######) was presented between each stimulus. Participants were simply instructed to make a button press response to any word that described a profession, of which there were 8, quasi-randomly distributed throughout the set (ie. two profession words in each quarter). The acquisition phase duration was approximately 7 minutes. The surprise recognition phase began approximately 45 minutes after the acquisition phase. In the intervening period, participants completed the structural imaging and exited the MRI scanner. In the recognition phase (performed at a desktop PC), 140 words (the 80 words seen previously, plus 60 new words) were presented in random order. Each word was presented individually for 1 second followed by a mask. Participants were instructed to respond to each word using button 1 (‘old’) or 2 (‘new’) on the PC’s numeric keypad, to indicate whether they thought it was previously studied in the acquisition phase (Fig. 1) Once a response was recorded, the next word was presented. The words used in both the acquisition and recognition phases were drawn from the MRC psycholinguistic database (RRID:SCR_014646) (http://www.psych.rl.ac.uk/MRC_Psych_Db.html) and matched for lexico-semantic features of length (all words employed were 6 letters long), frequency, familiarity and imageability, according to Kucera-Francis norms, as this can impact recognition performance^60^.

### MR Imaging and analysis

All imaging data was acquired with a Siemens Avanto 1.5 T scanner (Siemens Erlangen, Germany) using a 32-channel head coil and gradient strength 44 mT/m.

#### Volumetric analyses

A 3D T1-weighted MPRAGE sequence provided the anatomical images (5 minutes): parameters were TR = 1160 ms, TE = 4.44 ms, TI = 600 ms,FOV = 230 × 230 mm, acquisition matrix = 256 × 256, flip angle = 15°, with a resulting voxel size of 0.9 × 0.9 × 0.9 mm. Freesurfer 6.0 was used for cortical reconstruction and volumetric segmentation (http://surfer.nmr.mgh.harvard.edu). The Freesurfer reconstruction pipeline normalizes, motion corrects, and excludes non-brain tissue via a hybrid watershed/surface deformation procedure before transforming the images into Talairach space and segmenting them^61,62^. Hippocampal volumes were calculated using the automated hippocampal and amygdala segmentation algorithm included with the development version of FreeSurfer 6.0 (dev-20190118), which uses a probabilistic atlas built with ultra-high resolution MRI data to segment the hippocampal substructures and nuclei of the amygdala. This offers an advantage over the hippocampal module released with FreeSurfer 6.0 since joint segmentation of hippocampus and amygdala ensures a more accurate estimate of hippocampal volume^63,64^. To account for differences in head size, we normalized the calculated volume of left and right hippocampus by dividing by the estimated total intracranial volume (from the Freesurfer segmentation) to give a volume ratio.

#### NODDI

The following acquisition parameters were used for the diffusion-weighted imaging: TR = 8400 ms, TE = 99 ms, 60 slices, matrix size = 96 × 96, FOV = 240 × 240 mm^2^, resulting in voxel dimensions 2.5 × 2.5 × 2.5 mm^3^. Three diffusion-weighted shells were acquired using b = 300, 800, and 2400 s/mm^2^ with 9, 30 and 60 unique non-colinear diffusion directions, respectively. In addition, 11 non-diffusion-weighted volumes (i.e. b ≈ 0) were acquired. The total acquisition time was 15 minutes. The diffusion-weighted data were corrected for movement and eddy current distortion using a tool provided as part of FSL (FMRIB Software Library v5.0; http://fsl.fmrib.ox.ac.uk/fsl). Using the NODDI toolbox for MATLAB (Zhang 2012; http://www.nitrc.org/projects/noddi_toolbox) a NODDI microstructural model was fitted to the data. This calculated neurite density index (NDI), ODI, and free water volume fraction (fiso) in native space. Using ANTS software (http://stnava.github.io/ANTs/)parameter maps for each of these were normalized into MNI space. First an optimal brain template was generated based on the mathematical average of all participants’ mean b0 images (from the NODDI acquisition) using a maximum of 30 × 90 × 20 interactions per registration using the greedy-syn transformation model and cross-correlation estimation to assess similarity. This process also provided the diffeomorphic transformation required to deform the native-space brain images to the study-specific template space. Next, the diffeomorphic transformation required to warp the study-specific template to standard MNI space was calculated and both transformations (native-group and group template-MNI) were combined so that each brain was warped to MNI space in a single step. Finally, binarized masks for left and right hippocampal grey matter (regions labelled ‘hippocampus’ in the Harvard-Oxford Subcortical atlas) were applied to the ODI parameter maps using FSLmaths. ODI ranges from 0 (no dispersion of the fibre orientations) to 1 (fully dispersed).

#### Statistical comparisons

For all structural data, between-genotype differences were assessed using ANOVA in SPSS 25. Data met all assumptions for the use of these tests; gender was included as covariate in all analyses. To assess correlations with task performance, Spearman’s rank correlation coefficients were calculated. To test for differences in correlations between groups, a frequentist approach was taken using a percentile bootstrap method^65^. After 2000 iterations, the distribution of bootstrap correlation differences was assessed to determine significance. This analysis was performed in Matlab using code adapted from https://github.com/GRousselet/blog/tree/master/comp2dcorr and is more robust than other approaches^66^. Volumetric differences were assessed separately for left and right hippocampal formations. NODDI data were averaged across left and right, for consistency with the ROI used by Nazeri *et al*.^54^.

#### fMRI

The fMRI acquisition and preprocessing protocol has been described previously^67^: fMRI datasets were acquired using a T2* -weighted 2D-EPI sequence. To minimise signal artefacts originating from the sinuses, axial slices were tilted 30° from inter-commissural plane. Thirty-six 3 mm slices were acquired (0.75 mm interslice gap, interleaved (ascending) acquisition, phase encoding direction = A/P) with a matrix size of 64 and an in-plane resolution of 3 mm × 3 mm (TR = 3300 ms per volume, TE = 50 ms, flip angle = 90°, FoV = 192 mm). A total of 118 volumes were acquired (including 5 dummy volumes) yielding a total acquisition time of ~6.5 minutes. Field maps (phase and magnitude images) were also acquired for use in the unwarping stage of data preprocessing. Participants were positioned comfortably within the head coil; foam padding around the head minimized head movements. The experimental protocol was viewed on a screen via a mirror attached to the head coil. An MR compatible button box was used to collect responses: participants indicated a ‘sort’ detection using the first finger of their right hand.

Images were pre-processed using SPM8 (RRID:SCR_007037) (http://www.fil.ion.ucl.ac.uk/spm/). Default settings were used throughout. After discarding the first 5 volumes, the remaining volumes were spatially realigned to correct for head motion, and unwarped. A mean image created from the realigned volumes was spatially normalized into standard stereotaxic space (at 3 × 3 × 3 mm^3^) using the Montreal Neurological Institute (MNI) template in SPM8. The derived spatial transformation was then applied to the realigned and unwarped volumes, which were finally spatially smoothed to facilitate group level statistics with a Gaussian kernel of 8-mm FWHM. fMRI data were analysed with the standard hierarchal model approach employed in SPM using methods described previously^17^. Design matrices were constructed for each participant, that modelled subsequently remembered (Number of trials ranged from 19 to 54), subsequently forgotten (Number of trials ranged from 18 to 53), and profession sort trials as separate regressors. Movement parameters were also entered. Processing of fMRI data was performed blind to group membership. Contrasts for subsequently remembered and forgotten trials were entered at the first level, and effects of condition (remembered/forgotten) and genotype (e4−/e4+) were analysed at the second level using a flexible factorial to test for the effects of condition and condition by genotype interaction, followed by a 2 sample t-test (with the 2 conditions averaged) to test for main effects of genotype. Gender was entered as a covariate.

For main task effects, correction was performed over the whole brain. For genotype effects, given our a priori hypothesis, a small volume correction was applied using a mask defined according to the automated anatomical labeling atlas (AAL) from the Wake Forest University PickAtlas toolbox (RRID:SCR_007378)^68^. This mask comprised the bilateral hippocampi and parahippocampi. Localisation of clusters was performed using the SPM Anatomy Toolbox (RRID:SCR 013273)^69^. For all comparisons, a voxel-wise FWE-corrected threshold of p < 0.05 was applied.

## Results

### Behaviour: acquisition phase – categorisation performance

Participants showed high levels of accuracy when identifying the 8 profession words (see Table 2). The number of false alarms was low: Mean = 0.87, sd = 1.12. There were no effects of genotype on categorisation performance or false alarms.

**Table 2:** Proportion correct and s.d. for the 8 Categorisation trials at acquisition. Proportion (and s.d.) of Hits, Misses, False Alarms and Correct Rejections during the recognition phase, and sensitivity (d prime).

	Acquisition: Categorisation	Hits	Misses	False Alarms	Correct Rejections	d prime
e4− (n = 16)	0.93 ± 0.07	0.59 ± 0.11	0.41 ± 0.14	0.30 ± 0.11	0.70 ± 0.14	0.80 ± 0.38
e4+ (n = 16)	0.92 ± 0.05	0.62 ± 0.10	0.37 ± 0.11	0.33 ± 0.08	0.67 ± 0.08	0.80 ± 0.38

### Behaviour: recognition phase

See Table 2. Responses were classified as ‘Hits’ (‘Old’ words correctly identified) and ‘Correct Rejections’ (‘New’ words correctly identified). There was no effect of genotype on ‘Hits’ (F(1,31) = 0.567, p = 0.457) or ‘Correct Rejections’ (F(1,31) = 0.747, p = 0.394). To assess sensitivity according to signal detection theory, d prime was calculated using the Hit rate and False Alarm (‘New’ words incorrectly identified as ‘Old’) rate. There was no significant difference in d prime between the genotype groups (F(1,31) = 0.001, p = 0.982).

### Neuroimaging data

#### Structural: hippocampal volumes

**Figure 2:**
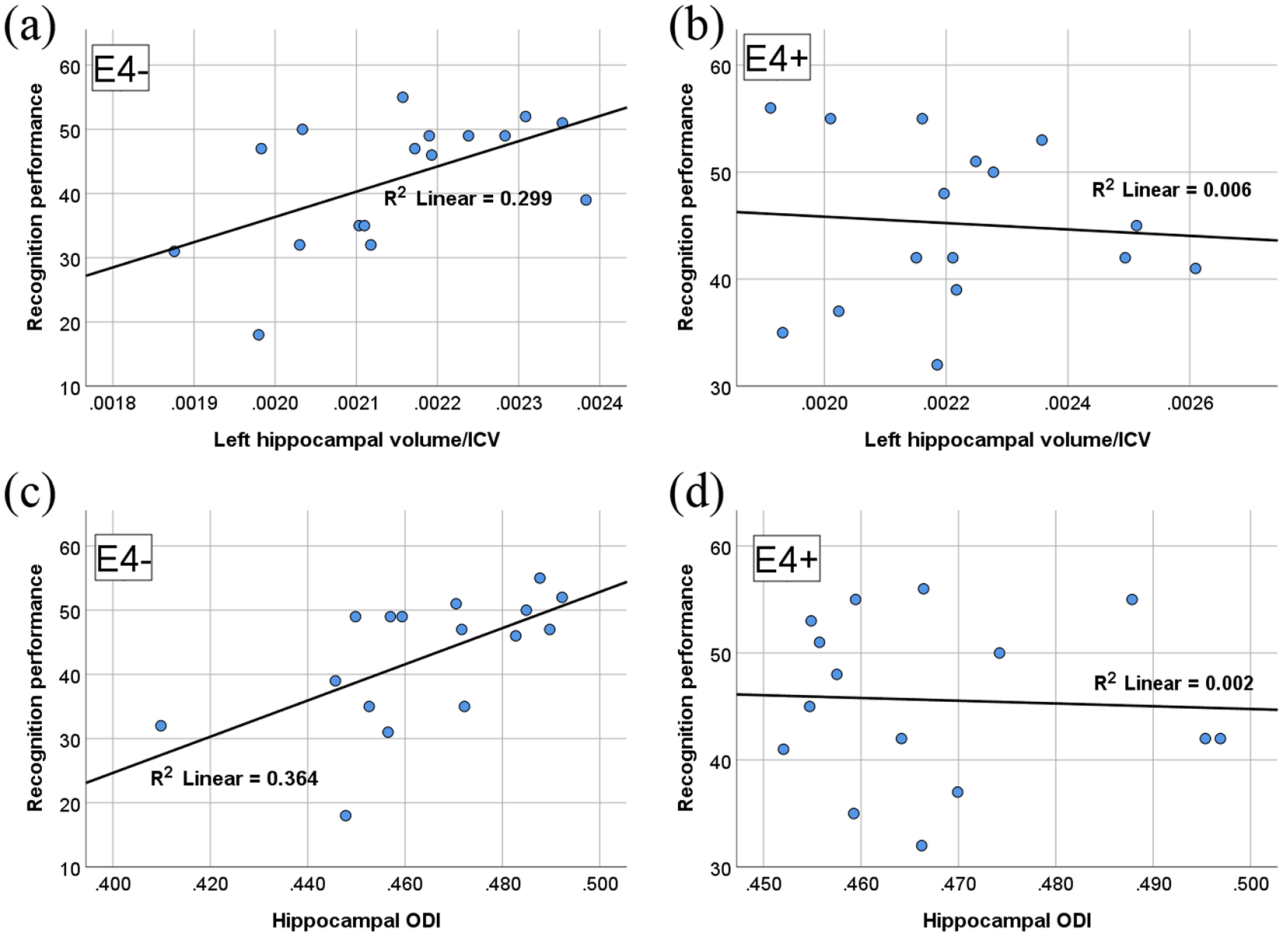
Correlations between recognition performance (number of ‘old’ words correctly recognised) and ICV-corrected left hippocampal volume in (**a**) e4− and (**b**) e4+. Correlations between recognition performance and hippocampal ODI in (**c**) e4− and (**d**) e4+.

No significant differences in hippocampal volumes (from Freesurfer) were observed between genotype groups (Table 3) for left (F(1,31) = 1.390, p = 0.247) or right (F(1,31) = 3.063, p = 0.090) hippocampus. In e4−, a significant positive correlation was seen between left hippocampal volume and proportion of ‘old’ words correctly identified at recognition (ρ = 0.586, p = 0.0135), Fig. 2(a). This was not the case in e4+ (ρ = −0.066, p = 0.807), Fig. 2(b). Employing a frequentist approach, the difference between the two correlation coefficients was found to be significant (p = 0.047, 1-tailed); a 1-tailed test was applied in line with our *a priori* hypotheses.

**Table 3:** Hippocampal volumes by genotype as a percentage of total Intracranial Volume (ICV). Correlations (Pearson’s r and associated p value) between volumes and proportion of ‘Old’ words correctly identified in the recognition phase.

	Left Hippocampus		Right Hippocampus	
	Volume (%ICV), s.d.	Correlation with %remembered	Volume (%ICV), s.d.	Correlation with %remembered
e4− (n = 16)	0.215 ± 0.0145	r = 0.547 (p = 0.023)	0.2183 ± 0.0142	r = 0.208 (p = 0.422)
e4+ (n = 16)	0.2219 ± 0.0201	r = −0.079 (p = 0.771)	0.2299 ± 0.0229	r = −0.169 (p = 0.531)

#### Structural: NODDI

ODI was calculated from an ROI encompassing bilateral hippocampal regions in line with Nazeri *et al*.^54^. No significant genotype differences were observed (F(1,31) = 0.237, p = 0.630) (Table 4). In e4− only, a significant positive correlation was seen between ODI and correct identification of ‘old’ words at recognition (ρ = 0.622, p = 0.010), Fig. 2(c). There was no relationship in e4+ (ρ = 0.004, p = 0.990), Fig. 2(d) and a significant genotype difference between the two correlation coefficients was confirmed using a frequentist approach (p = 0.0240, 1-tailed).

**Table 4:** NODDI results. ODI for bilateral hippocampal regions, by genotype. Correlations (Pearson’s r and associated p value) between ODI and percentage of ‘Old’ words correctly identified in the recognition phase.

	Mean ODI, s.d.	Correlation with %remembered
e4− (n = 16)	0.4644 ± 0.02153	r = 0.603 (p = 0.013)
e4+ (n = 16)	0.4676 ± 0.01476	r = −0.050 (p = 0.861)

#### fMRI: Subsequent Memory Task (Acquisition)

**Figure 3:**
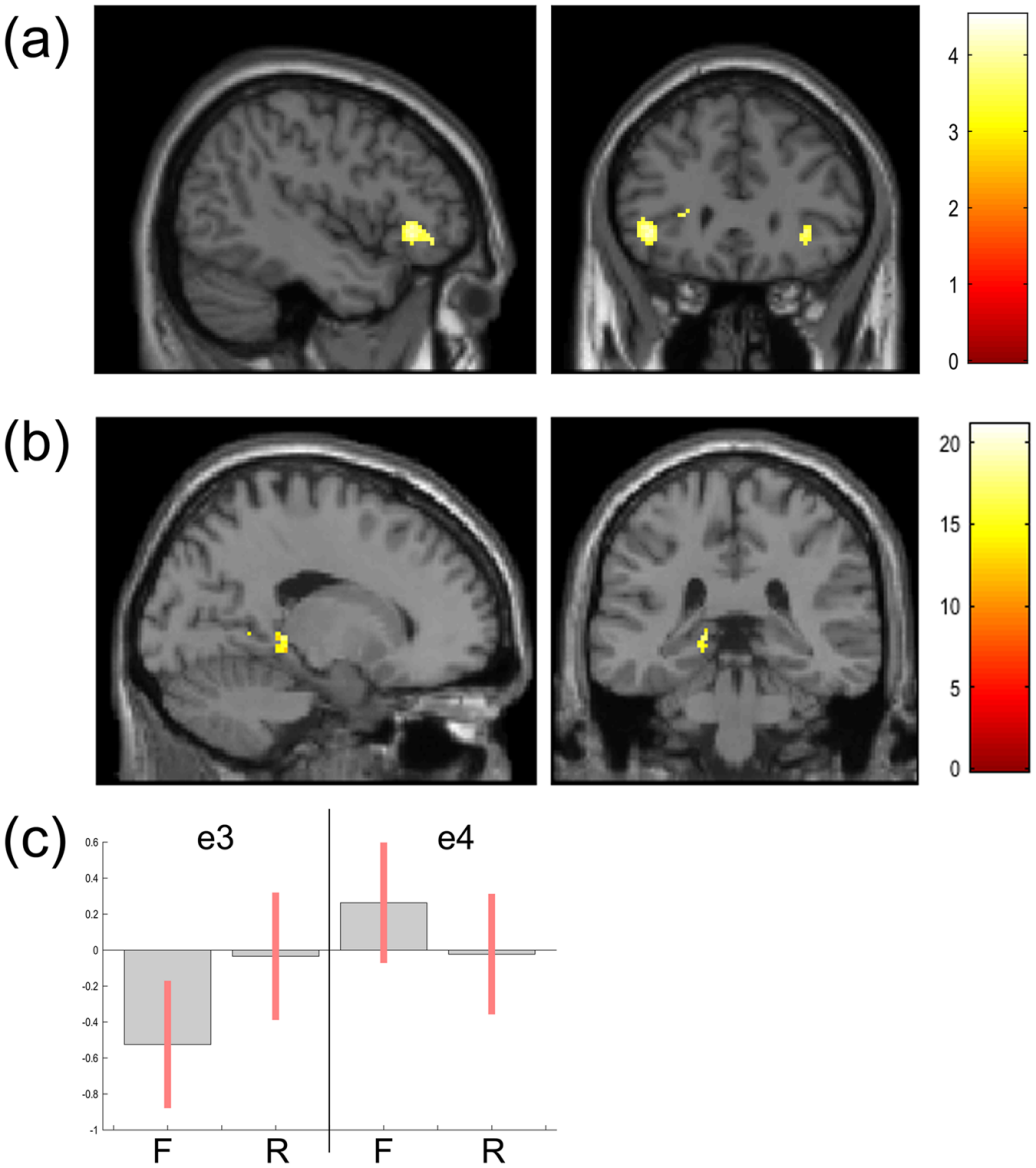
Activation maps (at p < 0.001 uncorrected) showing (**a**) Greater left IFG activity to subsequently remembered over forgotten trials in all subjects (**b**) Interaction with genotype in left parahippocampus (subiculum) after SVC and (**c**) the associated parameter estimates with 90% CI (F = Forgotten, R = Remembered) separately for e4− (e3) and e4+ (e4).

Remembered > Forgotten: Across all volunteers, significantly greater activation was seen in left inferior frontal gyrus (IFG) to subsequently remembered over forgotten trials (Table 5, rendered in Fig. 3a).

**Table 5:** Acquisition phase: fMRI results by contrast (a voxel-wise FWE-corrected threshold of p < 0.05 was used).

Contrast	Region	Vox	x, y, z	P value
Remembered > Forgotten (all subjects)	Left IFG	129	−44, 30, −4	P = 0.038 FWE-corrected
Condition by genotype interaction (all subjects)	Left Parahippocampus (subiculum)	4	−16, −34, 0	P = 0.013 FWE-corrected after bilateral S.V.C.
Remembered > Forgotten (e4− only)	Left IFG	180	−44, 28, −4	P = 0.007 FWE-corrected
	Left Parahippocampus (subiculum)	18	−18, −34, 4	P = 0.023 FWE-corrected after bilateral S.V.C
Remembered > Forgotten (e4+ only)	Left IFG	88	−44, 29, −4	P = 0.047 FWE-corrected

##### Effects of genotype

There was no main effect of genotype. Further, the directional contrasts e4+ > e4−, e4+ < e4−, over both conditions (remembered/forgotten) showed no effects at the whole brain level, or after small volume correction (SVC) with the bilateral hippocampus/parahippocampus ROI mask.

##### Condition by genotype interaction

Significant activity was observed in left hippocampus, after SVC for bilateral parahippocampus/hippocampus (Table 5, rendered in Fig. 3b, parameter estimates and 90% CI in Fig. 3c). The significant cluster was localised to the subiculum using the Anatomy toolbox. Parameter estimates suggested that this interaction was driven by greater activity to remembered over forgotten items in e4− only (Fig. 3c). This was tested by applying the contrasts (Remember > Forgotten) separately for each genotype group. In e4−, significant levels of Remember > Forgotten activity were observed in left IFG, and in left parahippocampus (after SVC), again localised to the subiculum using the Anatomy toolbox. In e4+, the same Remember > Forgotten contrast revealed activity in IFG only (Table 5).

## Discussion

In this study we set out to comprehensively explore, in a group of mid age volunteers, *APOE* effects on MTL structure and function during subsequent memory acquisition. A consistent pattern of MTL disruption was identified in e4+. A blunted functional response and a breakdown in the relationship between MTL structure and memory performance was evident. Mid age represents a critical but understudied age point, and these findings advance knowledge of the possible mechanisms underlying enhanced risk of AD and cognitive decline in e4+.

Previous studies suggest a pattern of MTL dysregulation in e4+ evident even in the absence of cognitive differences. In terms of functional response, we found a genotype by condition interaction in the left hippocampal formation, with activity in e4+ failing to differentiate subsequently remembered from forgotten items. There was no main effect of genotype, suggesting a specific disruption of memory-related MTL activity in e4+, rather than a more general pattern of over- or under- activity. Nevertheless, recall performance was equal between the genotype groups, suggesting that at this age point these disrupted MTL activity patterns do not significantly impact performance. These activation differences could signify a vulnerability marker and this finding accords with and adds to the literature showing MTL activation differences in e4+. While young adult e4+ often show MTL hyperactivation^13,16,17^, hypoactivation is reported with increasing age^24^. A major strength of the current study is the narrow 45–55 year age range: we focussed on the point in life where processes important for AD risk might be detectable (and potentially modifiable), but before the onset of significant cognitive decline. Indeed, all volunteers had cognitively normal MoCA scores, and there were no recall performance differences between groups. In a previous study using a similar task, young adult e4+ (mean age 21) showed greater left MTL activity to subsequently recalled words, relative to e4− peers^17^. The current findings suggest that by mid age this difference is lost, with left MTL activity in e4+ no longer differentiating subsequently recalled from forgotten words. Some authors have suggested that e4+ MTL overactivity in youth might reflect compensation for early pathology^20^. Alternatively, overactivation might predispose e4+ to pathological changes due to a higher associated metabolic burden^70,71^. The decreased patterns of activity with age, demonstrated here and elsewhere^24^, favour the latter explanation. At mid age, compromised MTL function in e4+ might reflect increased risk for age-related cognitive decline. Trivedi *et al*. found that e4+ aged 43–65 showed significantly less activation in hippocampal regions to novel versus familiar words in the recognition phase of a subsequent memory task. Although (as here) there was no cognitive or MTL volumetric differences between the genotype groups, verbal encoding ability correlated with activity in left anterior hippocampus in e4−, but not e4+. This points to disrupted MTL functional relationships in mid age e4+.

Here, further evidence suggesting MTL disruption and pathological changes in e4+ was shown by the structural-performance correlation analyses. While hippocampal volumes and microstructure metrics were comparable between genotypes, interesting differences emerged when structural-performance correlations were contrasted between genotypes. Left hippocampal volume showed a significant positive correlation with recall performance in e4− but appeared to be absent in e4+, and these correlations were significantly different between genotype groups. Links between hippocampal volume and memory performance have been demonstrated in healthy adults, particularly at mid-age and younger^41,72,73^ albeit not consistently. Evidence suggests that this linkage might become increasingly unreliable with age: Rajah *et al*.^74^ found that hippocampal volumes predicted context memory performance in young adults (mean age 24) though no such relationship was evident in an older group (mean age 68). In e4−, we found that left MTL volumes correlated with memory performance, whereas this relationship was completely absent in e4+. This absence suggests mid age e4+ show the characteristics of the older group reported by Rajah *et al*.^74^ and is consistent with evidence suggesting accelerated neuronal ageing in e4+^25–27^. It is possible that subtle MTL pathology in mid age e4+ might underlie these effects: early pathological changes not severe enough to undermine cognition or produce a group effect on structural volume, but able to disrupt volume-function relationships.

Microstructure in the MTL was studied using NODDI. NODDI is an emerging technique and this is the first published study to use it to study *APOE* effects in healthy individuals. Previous work has relied on DTI techniques, but compared to DTI, NODDI is a better-specified model that provides additional microstructural measures. Here we were specifically interested in how ODI in hippocampal regions might be affected by e4 status. This was motivated by the findings of work by^54^ who investigated effects of age on grey matter neuritic organization and density, and relationship with cognitive performance. In healthy adults aged 21–84, a mediation analysis showed that higher hippocampal ODI was protective against cognitive aging. No other regions or diffusion measures showed this relationship. This could reflect extension and growth of the hippocampal dendritic tree as a compensatory response to partial deafferentation, and a marker of successful cognitive aging. Since e4+ are at higher risk of *un-*successful cognitive aging, we hypothesised that hippocampal ODI might be compromised in e4+, or fail to show a positive relationship with cognitive performance. Although no genotype differences were found, a disrupted relationship with performance was observed in e4+, analogous to the e4+ effects on volume-function relationships discussed above. Again, a positive relationship between ODI in hippocampal regions and recall performance was observed in e4− (consistent with higher hippocampal ODI exerting a protective effect on cognition^54^), but this relationship was absent in e4+. The difference in correlations was significant. Therefore in e4+ this neuroprotective mechanism, thought to be an important mediator of successful cognitive ageing, would appear to be non-functional. This is a novel and important finding. A failure to utilise greater dendritic branching to support memory performance highlights MTL vulnerability in e4+, and could contribute to the enhanced risk of age-related cognitive decline and AD. Further, the link between hippocampal ODI and cognitive performance in e4− adds valuable support to the findings of Nazeri *et al*.^54^ and justifies follow-up work based on these findings.

The number of participants (N = 16 per genotype group) should be noted as an important limitation of the present study. Although we employed robust statistical tests, the findings (especially those based on differences between correlations) should be interpreted with caution pending replication. Unfortunately, small sample sizes are all too common in the APOE literature. Future work should aim to replicate these findings in an expanded sample and also investigate e4 gene dose effects Use of connectivity-based approaches would also be beneficial so as to explore how the MTL disruption identified here interacts with other brain regions and networks.

In conclusion, we present a consistent set of findings in mid age e4+ that point to dysregulation of activity in the hippocampal formation, and disrupted relationships between structure and cognitive performance. The current work reinforces previous findings but add novel insights into possible mechanisms. A lack of hippocampal functional response in differentiating encoding success from failure was seen during memory acquisition in e4+. Hippocampal volumes correlated with memory performance in e4− but not e4+, suggesting that in e4+ this relationship might be disrupted by the presence of subtle early pathology. In terms of microstructure, hippocampal neurite orientation-dispersion correlated with better recall in e4− but again this relationship was absent in e4+. The inability to use this potential compensatory mechanism to support cognition could be an important contributor to enhanced risk of age-related decline and AD in e4+. This paper therefore provides novel insight into possible mechanisms linking APOE e4 to cognitive decline and AD.

## Data Availability

All extracted data upon which these analyses were based will be made available via Figshare. Due to confidentiality regarding the genotype data, the raw neuroimaging data cannot be made publicly available.
